# Development of an alginate-Matrigel hydrogel system to evaluate cancer cell behavior in the stiffness range of the bone marrow

**DOI:** 10.3389/fbiom.2023.1140641

**Published:** 2023-06-02

**Authors:** Logan A. Northcutt, Alyssa M. Questell, Julie Rhoades, Marjan Rafat

**Affiliations:** 1 Program in Cancer Biology, Vanderbilt University, Nashville, TN, United States,; 2 Department of Biomedical Engineering, Vanderbilt University, Nashville, TN, United States,; 3 Department of Clinical Pharmacology, Vanderbilt University, Nashville, TN, United States,; 4 Department of Chemical and Biomolecular Engineering, Vanderbilt University, Nashville, TN, United States,; 5 Department of Radiation Oncology, Vanderbilt University Medical Center, Nashville, TN, United States

**Keywords:** breast cancer, bone metastasis, bone marrow, biomimetic, hydrogel

## Abstract

Bone metastasis is highly prevalent in breast cancer patients with metastatic disease. These metastatic cells may eventually form osteolytic lesions and affect the integrity of the bone, causing pathological fractures and impairing patient quality of life. Although some mechanisms have been determined in the metastatic cascade to the bone, little is known about how the mechanical cues of the bone marrow microenvironment influence tumor cell growth and invasion once they have homed to the secondary site. The mechanical properties within the bone marrow range from 0.5 kPa in the sinusoidal region to 40 kPa in the endosteal region. Here, we report an alginate-Matrigel hydrogel that can be modulated to the stiffness range of the bone marrow and used to evaluate tumor cell behavior. We fabricated alginate-Matrigel hydrogels with varying calcium sulfate (CaSO_4_) concentrations to tune stiffness, and we demonstrated that these hydrogels recapitulated the mechanical properties observed in the bone marrow microenvironment (0.7–16 kPa). We encapsulated multiple breast cancer cell lines into these hydrogels to assess growth and invasion. Tumor cells in stiffer hydrogels exhibited increased proliferation and enhanced elongation compared to lower stiffness hydrogels, which suggests that stiffer environments in the bone marrow promote cellular invasive capacity. This work establishes a system that replicates bone marrow mechanical properties to elucidate the physical factors that contribute to metastatic growth.

## 1 Introduction

Metastasis, the spread of cancer cells from the primary tumor site to a distant site in the body, is a well-studied hallmark of cancer and is associated with higher death rates in patients (Hanahan and Weinberg, 2011; Chopra and Davies, 2020). In breast cancer, distant recurrence is common in sites such as the brain, bone, liver, and lungs (Berman et al., 2013). It has been shown that bone metastases are correlated with lower incidences of death in breast cancer patients, but 70% of patients who succumb to the disease have bone metastases after autopsy (Macedo et al., 2017). The bone marrow microenvironment is highly dynamic and composed of many fibrous macromolecules and progenitor cells (Buenrostro et al., 2014; Haider et al., 2020). The bone marrow is highly variable in terms of biophysical properties such as stiffness, three-dimensional (3D) architecture, and fluid flow. The process by which mechanical cues, such as matrix stiffness and porosity, can affect biophysical and biochemical responses is called mechanotransduction and is highly integral to tumor cell progression in the primary site (Martino et al., 2018). The mechanical forces of the environment can affect biological processes in the bone marrow such as the production of osteoactive agents for tumor-induced bone disease (Allen et al., 2012). Studies have shown that the stiffness of the bone marrow microenvironment can influence progenitor cells that are responsible for the development and prevalence of hemopoietic stem cells (Choi and Harley, 2017). Additionally, previous work has shown that the increased stiffness in the primary breast tumor microenvironment alters cell behavior, leading to more mesenchymal phenotypes and enhanced proliferation (Stowers et al., 2019). However, many sites of metastasis are ten-fold stiffer than the breast primary site (Choi and Harley, 2017), and how physical factors at metastatic sites influence tumor cell behavior is not well-studied. We therefore hypothesized that the stiffness changes within the bone marrow environment, which spans 0.3 kPa to >35 kPa (Nelson and Roy, 2016), will affect the behavior of cancer cells.

Like the breast microenvironment, systems to mimic the bone microenvironment can be synthetic or naturally derived (Northcutt et al., 2020). Cancer-related bone pathologies in both the marrow and bone are typically evaluated with *in vivo* models, which can be costly and can take time to see osteolytic effects, using techniques such as intratibial injections to study established tumors in bone as well as potential treatments to inhibit tumor-induced bone disease (Rosol et al., 2003; Blouin et al., 2008; Vanderburgh et al., 2019). Synthetic hydrogels are highly tunable but often require UV-crosslinking that may reduce the viability of encapsulated cells. Naturally-derived hydrogel systems can also be utilized for *in vitro* studies as they may better replicate the range of proteins and binding sites in tissues. In addition, engineered systems such as microfluidic devices have been used to study the metastatic properties of breast cancer to the bone matrix along with mineralized osteoblastic bone tissue (Dhurjati et al., 2008; Bersini et al., 2014).

Although many studies attempt to model the bone marrow and its surrounding environment, few systems can replicate its stiffness without changing the number of biological binding sites (Chaudhuri et al., 2014). Many of these materials evaluate stiffness in 2D environments, which limits studying the forces that surround the cell and potential interactions with the microenvironment (Baruffaldi et al., 2021; Xiao et al., 2022). 3D hydrogels typically do not incorporate both mechanical properties and biological complexity. Recently, Jansen et al. (2022) designed synthetic polyethylene glycol hydrogels with a bone marrow-specific protein signature to mimic the bone marrow microenvironment. This novel work combined relevant, tunable mechanical properties and chemical extracellular matrix (ECM) cues. However, the study focused on cell behavior in an environment that matched the average marrow modulus. Here, we present an alginate-Matrigel hydrogel system as a bone marrow model with varied crosslinking through calcium sulfate (CaSO_4_) to allow for changes in stiffness alone. While stiffness does not necessarily drive cell behavior in 3D (Riaz, 2016; Zonderland and Moroni, 2021), we are interested in probing how 3D environmental stiffness directly influences breast cancer cells, which may give insight into how sites of metastasis promote tumor growth and invasion. Indeed, the tunability of alginate and the ECM proteins that Matrigel provides allow for evaluating cellular mechanotransduction in 3D (Caliari and Burdick, 2016; Neves et al., 2020; Cao et al., 2021). We found the stiffness of our hydrogels can span two orders of magnitude within the range of the bone marrow microenvironment, which can alter tumor cell proliferation and morphology. Overall, we show how our tunable system may be used to understand how the stiffness of the bone marrow affects metastatic progression.

## 2 Materials and methods

### 2.1 Biomimetic hydrogel preparation

**FIGURE 1:**
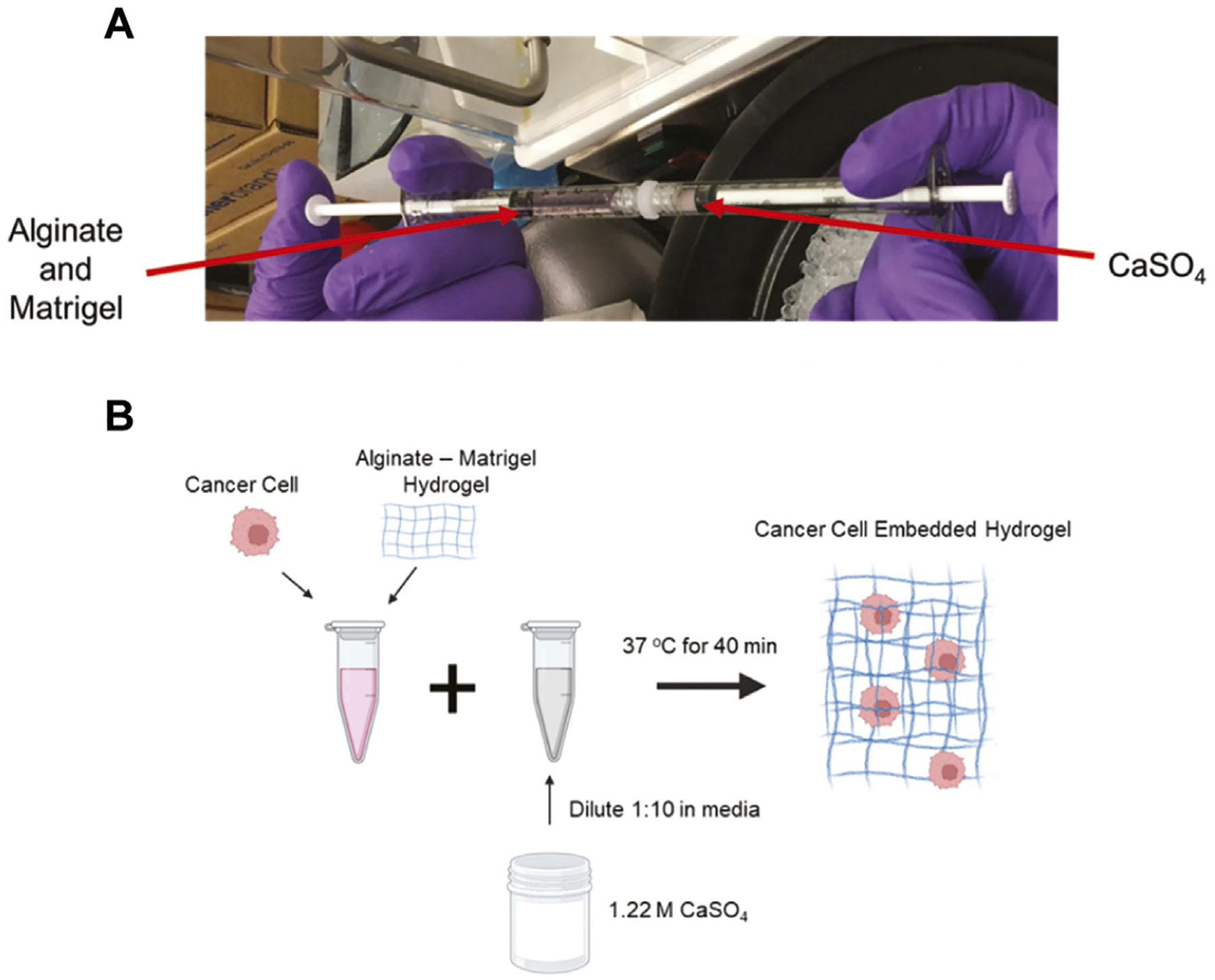
Development of crosslinked alginate-Matrigel hydrogels. (A) Image of component mixing to form hydrogels. (B) Schematic of cell encapsulation within hydrogels. CaSO_4_ concentrations from 5–50 mM were used to form the cell embedded hydrogels.

Hydrogels were developed as previously described (Chaudhuri et al., 2014; Wisdom and Chaudhuri, 2017). The hydrogels consisted of high G alginate (PRONOVA) 5–10 mg/mL and growth-factor reduced Matrigel (4.5 mg/mL, Corning). Calcium sulfate (CaSO_4_) was used for alginate crosslinking starting at a stock concentration at 1.22 M and diluted to a 122 mM working solution in the appropriate media. The volume of the working solution was adjusted for each condition to achieve a range between 5 and 50 mM CaSO_4_. To form hydrogels, two 1 mL syringes were connected via a Luer lock with alginate and Matrigel in one and CaSO_4_ in a separate syringe, and the solutions were mixed back and forth 10 times (Figure 1A).

### 2.2 Rheology measurements

Stiffness measurements of the hydrogels were conducted using a rheometer (AR 2000 Ex, TA Instruments) with a 25 mm top and bottom plate. The plate was rotationally mapped. 500 μL hydrogel solution was added to the plate, and a disk was formed by lowering the plate head. The plate was warmed to 37°C, and mineral oil was used to coat the edges of plate to prevent dehydration of the gel. The resulting plate separation was approximately 1,000 μm. Gel characteristics were measured over time until the storage modulus reached equilibrium (between 1 and 2.5 h depending on the crosslinking density) with 0.5% applied strain and strain frequency of 1 Hz. The average storage and loss modulus of the last 3 data points were averaged and calculated using the Young’s Modulus (E) equation in units of Pascals (Pa):

E=2G(1+ν)

where the ν is Poisson’s Ratio and assumed to be 0.5 (Chaudhuri et al., 2014).

G (bulk modulus) is calculated using G = (G’ + G″), where G′ is the storage modulus and G″ is the loss modulus.

### 2.3 Culturing of cancer cells in hydrogels

4T1 murine triple-negative breast cancer cells (ATCC) were cultured in RPMI media, supplemented with 10% heat-inactivated fetal bovine serum (HI-FBS) and 1% penicillin-streptomycin. MCF7 human estrogen receptor-positive breast cancer cells (from Dr. Rachelle Johnson, Vanderbilt University Medical Center) were cultured in DMEM media, supplemented with 10% HI-FBS and 1% penicillin-streptomycin. Cells were embedded at a concentration of 1.0 × 10^5^ cells/mL for each condition and incorporated in the hydrogel (Wisdom and Chaudhuri, 2017) (Figure 1B). This resulted in an evenly distributed single cell suspension. A MatTek dish with a No. 1 glass slide was used for culturing cells in hydrogels, and 100 μL of the cell-pre-gel solution was added to the wells for complete coverage (MatTek, P35G-0–10-C). Hydrogels were allowed to form for 45 min at 37°C before adding 3 mL of media to the wells. The cells formed clusters and were grown in the hydrogels for either 2 or 7 days in complete media. The media was changed every 2 days.

### 2.4 Fluorescence staining and cell imaging

Following culture, the gels were fixed in 10% formalin for 15 min and washed with phosphate-buffered saline (PBS) 3 times. The cells were permeabilized with 0.1% Triton in PBS and blocked with 5% normal goat serum (NGS) in PBS for 1 h. After blocking, 1000X Phalloidin (Phalloidin-iFluor 594 Reagent, Abcam) was diluted to 1X in 5% NGS and incubated for 1.5 h in the dark. After staining that actin cytoskeleton, the gels were mounted with Antifade Diamond Mount with NucBlue overnight. 10–15 images (0.045 mm^2^ field) per gel were acquired using a Leica DMi8 inverted fluorescence microscope. A minimum of 50 nuclei for the 2 days incubation or 150 nuclei for the 7 days incubation were counted per independent gel replicate.

### 2.5 Cluster morphology analysis

Using ImageJ, multicellular clusters were traced using the “Freehand Selection Tool” to measure the major and minor axis dimensions. The elongation index (EI) was calculated using the following equation (Cavo et al., 2018):

EI=Major Axis/Minor Axis

A minimum of 10 colonies for the 2 days incubation or 5 colonies for the 7 days incubation were evaluated per replicate.

### 2.6 Statistical analysis

Data were analyzed using analysis of variance (ANOVA) to determine statistical significance (*p* < 0.05) after confirming normality. All analyses were performed in GraphPad Prism 9.

## 3 Results and discussion

### 3.1 Alginate-Matrigel hydrogels crosslinked with CaSO_4_ replicate the stiffness of the bone marrow

**FIGURE 2:**
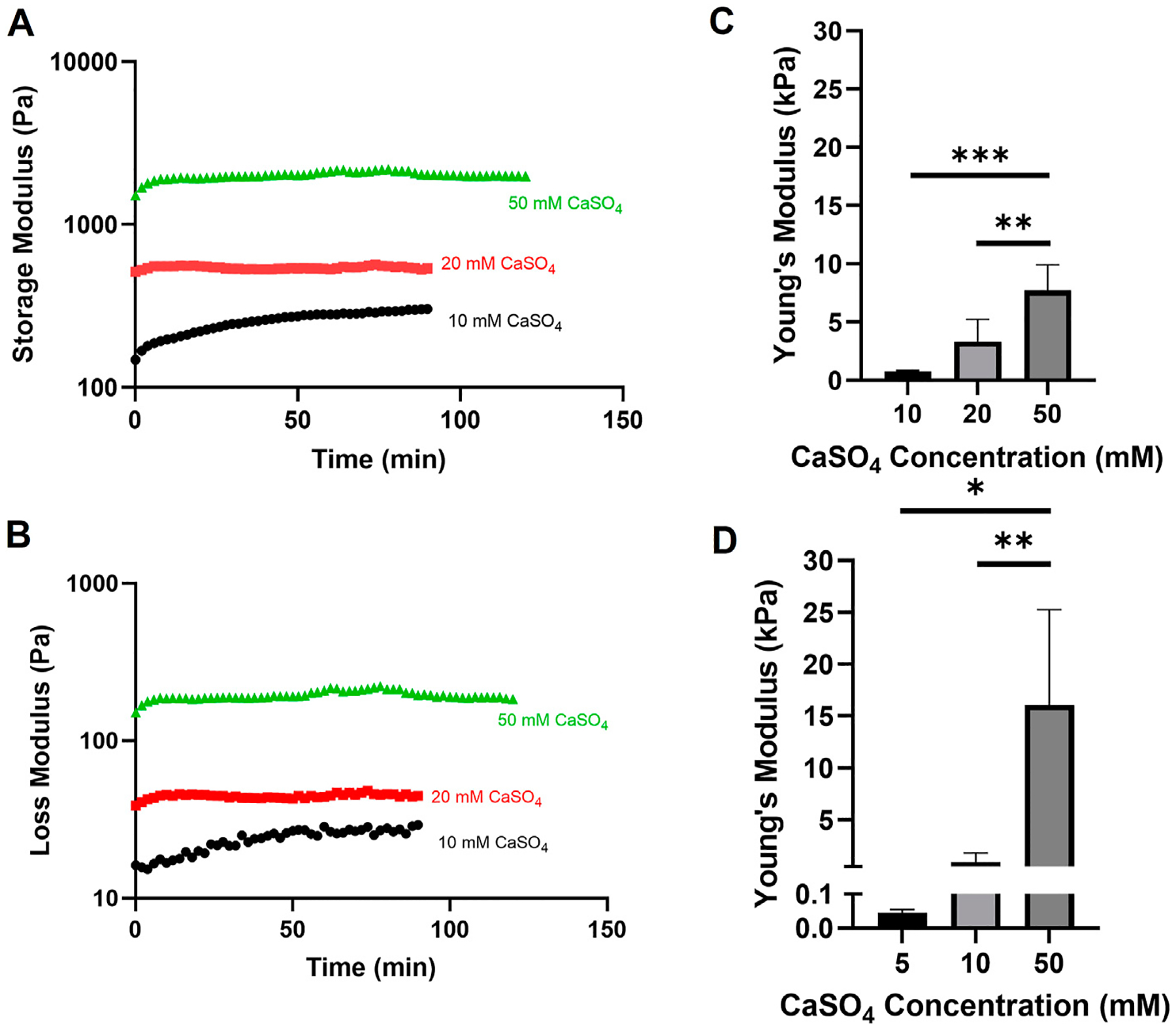
Evaluation of alginate-Matrigel hydrogel stiffness with varying alginate and calcium sulfate (CaSO_4_) concentrations. Hydrogel mechanical properties were analyzed using rheology. Time sweep analysis with the indicated crosslinker concentration is shown for the storage (A) and loss (B) modulus. Young’s moduli were calculated for 5 mg/mL (C) and 10 mg/mL (D) alginate-Matrigel hydrogels. Results represent *n* = 3–6 independent replicates. Error bars are standard deviation. Statistical significance was determined by ANOVA with *p < 0.05, **p < 0.01, and ***p < 0.001.

In previously published studies using the alginate-Matrigel system, the stiffness of the breast tumor microenvironment has been mimicked. Here, we intended to extend previously published methods by increasing CaSO_4_ concentrations up to 50 mM to achieve stiffnesses within the bone marrow microenvironment range of 0.3 to >35 kPa (Nelson and Roy, 2016). In hydrogels with 5 mg/mL alginate and 4.5 mg/mL Matrigel, Young’s moduli ranged from approximately 0.7–8 kPa when varying CaSO_4_ concentrations between 10 and 50 mM (Figures 2A–C). To increase the stiffness range, the alginate concentration was increased to 10 mg/mL, and the Young’s moduli range expanded to 16 kPa (Figure 2D). Although this system did not exceed 35 kPa, a stiffness range of more than two orders of magnitude was achieved, which to our knowledge has not been shown in similar alginate-Matrigel hydrogel systems. We continued to use the 5 mg/mL alginate formulation in our proof-of-concept studies, which spanned one order of magnitude within the stiffness range of the bone marrow, as the 10 mg/mL formulation showed reduced viability in our cell lines. Future studies will explore composite hydrogels and the addition of relevant peptides to the bone marrow microenvironment that may better support cell growth.

### 3.2 Evaluating tumor cell proliferation

**FIGURE 3:**
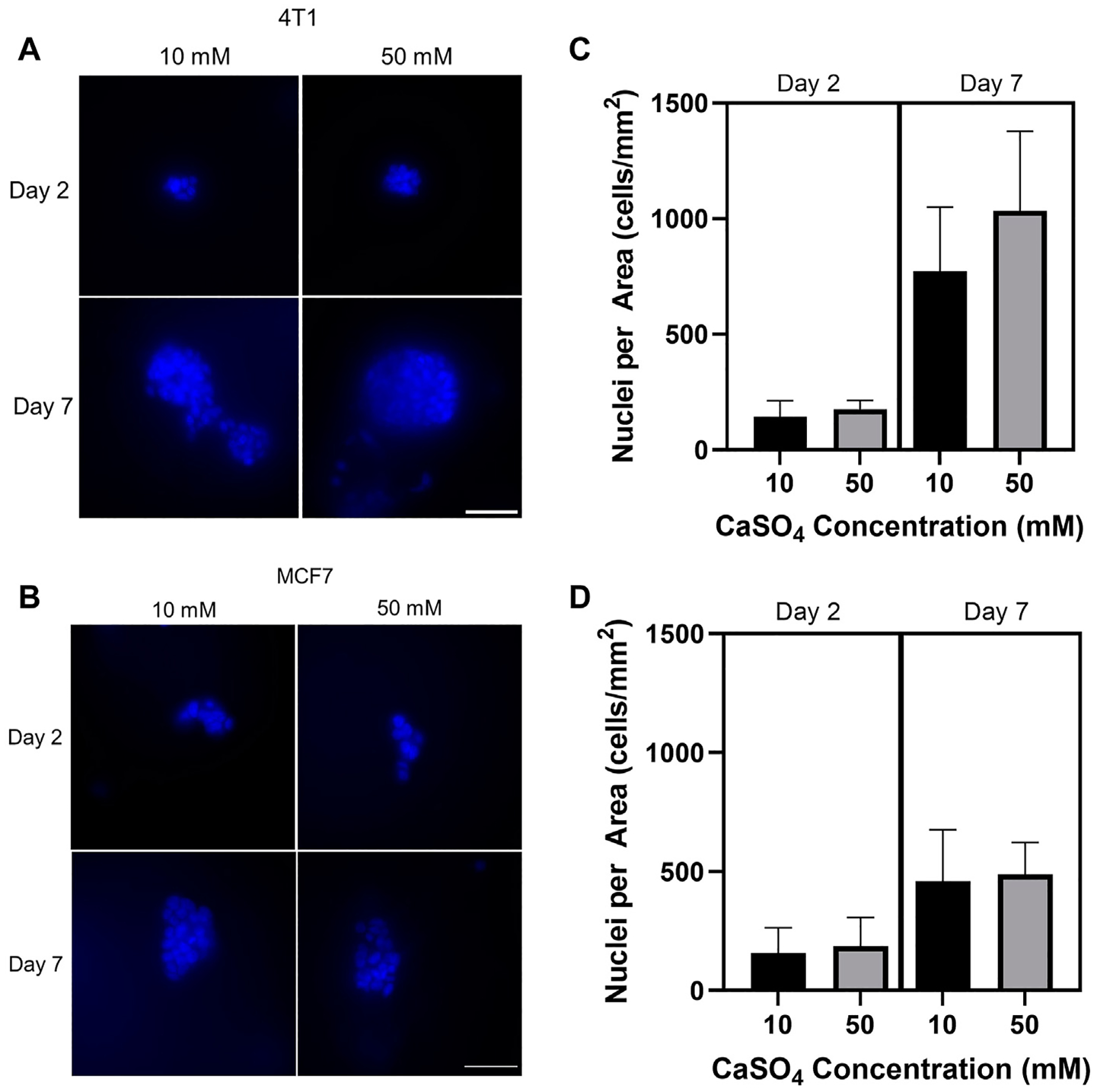
Determination of the effect of stiffness on breast cancer cell proliferation. Breast cancer cells (mouse 4T1; human MCF7) were embedded into alginate (5 mg/mL)-Matrigel hydrogels crosslinked with 10 and 50 mM CaSO_4_ up to 7 days, and stained nuclei (blue) were counted. Representative 4T1 (A) and MCF7 (B) images are shown. Quantification of nuclei for 4T1 (C) and MCF7 (D) using ImageJ. 10–15 fields of 0.045 mm^2^ were taken per independent hydrogel replicate (*n* = 3) to determine nuclei counts. Cells were seeded at a concentration of 1 × 10^5^ cells/mL. Scale bar is 50 μm. Error bars are standard deviation.

After developing hydrogels within the stiffness range of the bone marrow microenvironment, we then evaluated the proliferative response of tumor cells encapsulated in the hydrogels by counting the nuclei in cell clusters. 4T1 and MCF7 cells were seeded in 1 kPa (10 mM CaSO_4_) and 8 kPa (50 mM CaSO_4_) hydrogels up to 7 days. Fluorescence images from nuclear staining demonstrate an increasing proliferation trend in 4T1 cells (Figures 3A, C) but not MCF7 cells (Figures 3B, D) after 7 days. Both 4T1 and MCF7 cells are known to grow in clusters in 3D (Krause et al., 2010; Li and Lu, 2011; Perrin et al., 2022). Nuclei counts were used as a proxy for proliferation in this study, and we therefore need to further validate the results. Future studies will confirm proliferative capacity through Ki67 staining as well as additional proliferation assays that directly measure DNA synthesis. In addition, we will evaluate cell proliferation beyond 7 days, which may reveal larger differences in cell subtype.

### 3.3 Increased stiffness enhances elongation in breast cancer cell clusters

**FIGURE 4:**
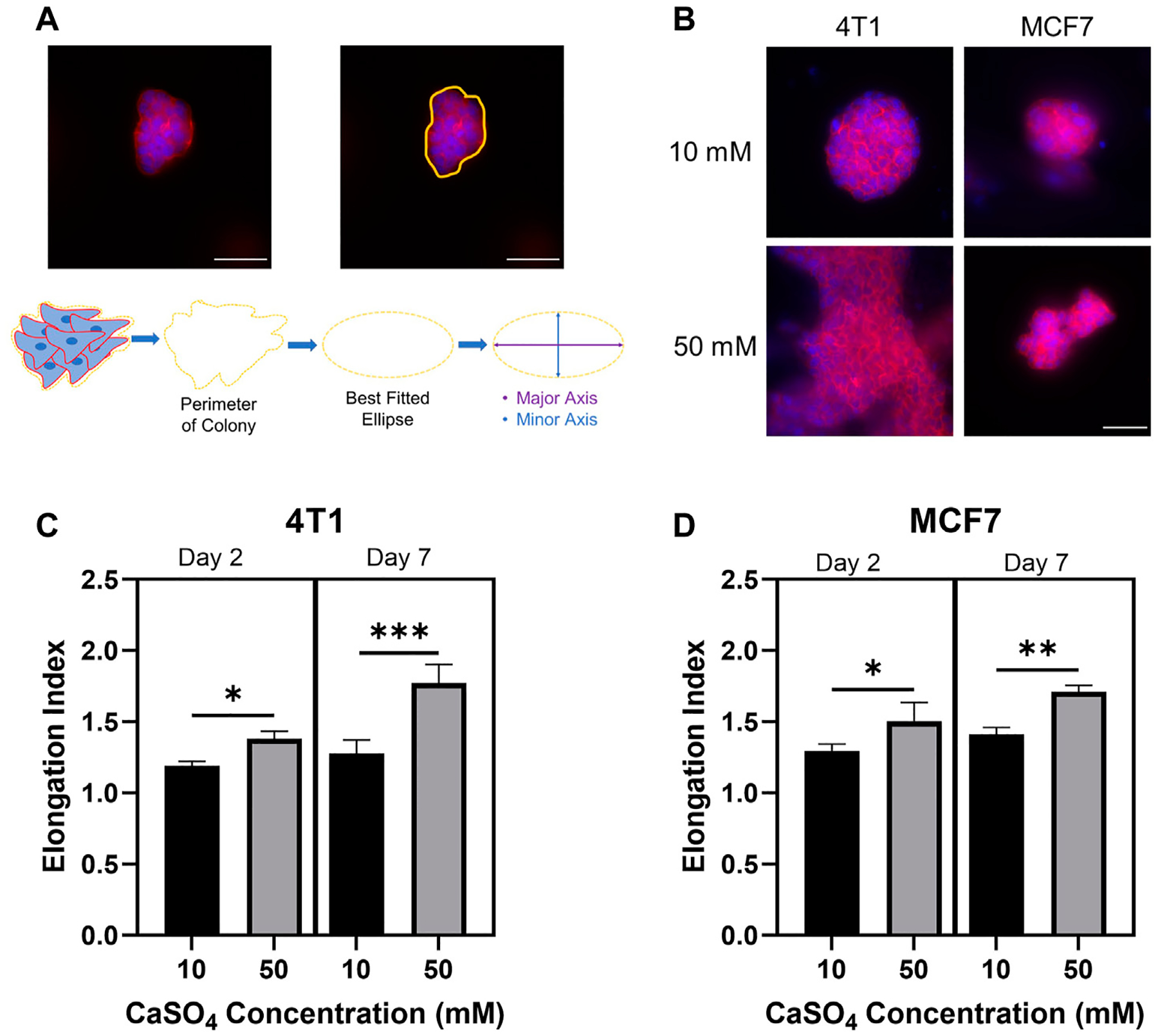
Investigating breast cancer cell cluster morphology to determine invasive capacity. (A) Example analysis of cell cluster elongation index. (B) Representative images of F-actin (red) and nuclei (blue) staining of 4T1 and MCF7 cells following 7 days incubation in alginate (5 mg/mL)-Matrigel hydrogels crosslinked with 10 mM and 50 mM CaSO_4_. Quantification of elongation index in 4T1 (C) and MCF7 (D) cell clusters following incubation for 2 and 7 days. Scale Bar is 50 μm. Results represent *n* = 3 independent hydrogel replicates with a minimum of 10 colonies evaluated for the 2 days incubation or 5 colonies for the 7 days incubation per replicate. Error bars are standard deviation. Statistical significance was determined by ANOVA with *p < 0.05, **p < 0.01, and ***p < 0.001.

Cell morphology has been shown to correlate with the ability of tumor cells to become invasive and motile (Anderson et al., 2006). Additionally, alginate-Matrigel systems mimicking breast tissue environments display increased cell elongation and expression of epithelial-to-mesenchymal transition markers (Stowers et al., 2019). However, this system has not been shown to mimic the bone marrow microenvironment. Here, we evaluated the morphology of encapsulated cell clusters by determining the EI following F-actin staining (Figures 4A, B). EI showed statistically significant increases after 2 and 7 days in both cell lines in stiffer hydrogels (Figures 4C, D). 4T1 cells showed a greater increase in EI following 7d compared to MCF7s with an approximately 40% increase in EI compared to a 20% increase in MCF7 cells in stiffer hydrogels. Taken together, we have shown the feasibility of studying breast cancer cell invasive properties and cytoskeletal dynamics in a biomimetic bone marrow hydrogel system and that there may be a differential response according to subtype. We measured cell cluster elongation consistent with invasion, but additional work must be done to evaluate cellular invasion beyond correlative properties. We will evaluate movement through the gel, invadopodia through cortactin-actin co-localization, and invasion gene signatures in the future.

### 3.4 Limitations

Although our work is useful for understanding cellular responses within the stiffness of the bone marrow, using confocal or two-photon microscopy will enhance the cellular image quality. Our study evaluates single stiffness gels, which do not capture the complex physical properties of the bone marrow. Future studies will expand this work to incorporate a stiffness gradient to replicate the bone marrow microenvironment more accurately. It is also necessary to study multiple microenvironmental factors in the bone marrow, including other mechanical cues. While this system directly evaluates stiffness, the primary components of Matrigel are laminin and collagen IV whereas the bone marrow is mainly comprised of collagen I (Boskey, 2013). Characterizing and mimicking the bone marrow ECM, controlling and modulating other physical properties such as degradability, and including additional relevant cell types will increase the impact of this system. Lastly, we visualized cells using fluorescent markers. Future studies will explore cell extraction from hydrogels to examine specific molecular mechanotransduction pathways involved in promoting metastasis in the bone marrow microenvironment.

## 4 Conclusion

Currently, there are limited systems that allow for varying environmental stiffnesses without changing the number of biological binding sites. We have developed alginate-Matrigel hydrogels that replicate the stiffness within the bone marrow microenvironment by changing only crosslinker concentration and leaving binding sites constant. This stiffness range has not been previously studied. Additionally, we have shown the possibility of studying proliferation and invasive capacity in this system. This proof-of-concept work will be expanded in the future to evaluate additional cell types, time points, and gene expression to better understand the role of mechanical properties in influencing metastatic potential in breast cancer.

## Data Availability

The raw data supporting the conclusion of this article will be made available by the authors, without undue reservation.
